# Symptomatic Common Carotid Artery Stenosis Managed With Carotid Endarterectomy

**DOI:** 10.7759/cureus.49062

**Published:** 2023-11-19

**Authors:** Max Murray-Ramcharan, Son-Isha Coetzee, Peter Patalano

**Affiliations:** 1 General Surgery, Harlem Hospital Center, New York, USA; 2 Surgery, St. George's University School of Medicine, New York, USA; 3 Vascular Surgery, Woodhull Medical Center, Brooklyn, USA

**Keywords:** stroke, transient ischemic attacks, carotid endarterectomy (cea), common carotid artery stenosis, common carotid artery

## Abstract

Extracranial carotid artery disease is typical at the carotid bifurcation and internal carotid artery (ICA) and is rarely symptomatic from isolated common carotid artery (CCA) stenosis. We present the case of a 60-year-old female patient who presented with a transient ischemic attack (TIA) with significant stenosis of the ipsilateral CCA only, without any involvement of the ICA or bifurcation. This was treated with carotid endarterectomy (CEA) with desirable postoperative outcomes; at up to six months postoperative follow-up, this patient had no recurrence of symptoms. We draw attention to the current gap in the literature with regard to a lack of specific guidelines for optimal evidence-based surgical treatment for this specific condition, with recent advances within certain vascular societies.

## Introduction

Extracranial carotid artery disease commonly forms at the carotid bifurcation with extension into the internal carotid artery (ICA) [1]. Typically, surgical intervention is reserved for patients with ICA or carotid bifurcation disease satisfying criteria outlined by the Society for Vascular Surgery clinical practice guidelines (2022) [2]. The associated neurologic symptoms, including transient ischemic attack (TIA) and stroke, sometimes seen in this condition are due to flow disturbances at the carotid bifurcation affecting cerebral blood flow [3], often with thromboembolic material from atherosclerotic plaques dispersed into the middle cerebral artery territory. As a result, symptomatic disease of the common carotid artery (CCA) is therefore uncommonly seen, with only a few notable cases reported in the literature and an estimated incidence of 2-4% in patients undergoing angiography for symptomatic cerebrovascular disease [4]. No specific guidelines are mentioned for the disease of the CCA, likely due to its relative rarity [5] in clinical practice. We present the case of a patient who presented with a left-sided TIA with findings of significant isolated stenosis of the distal left CCA and was successfully managed with carotid endarterectomy (CEA).

## Case presentation

**Figure 1:**
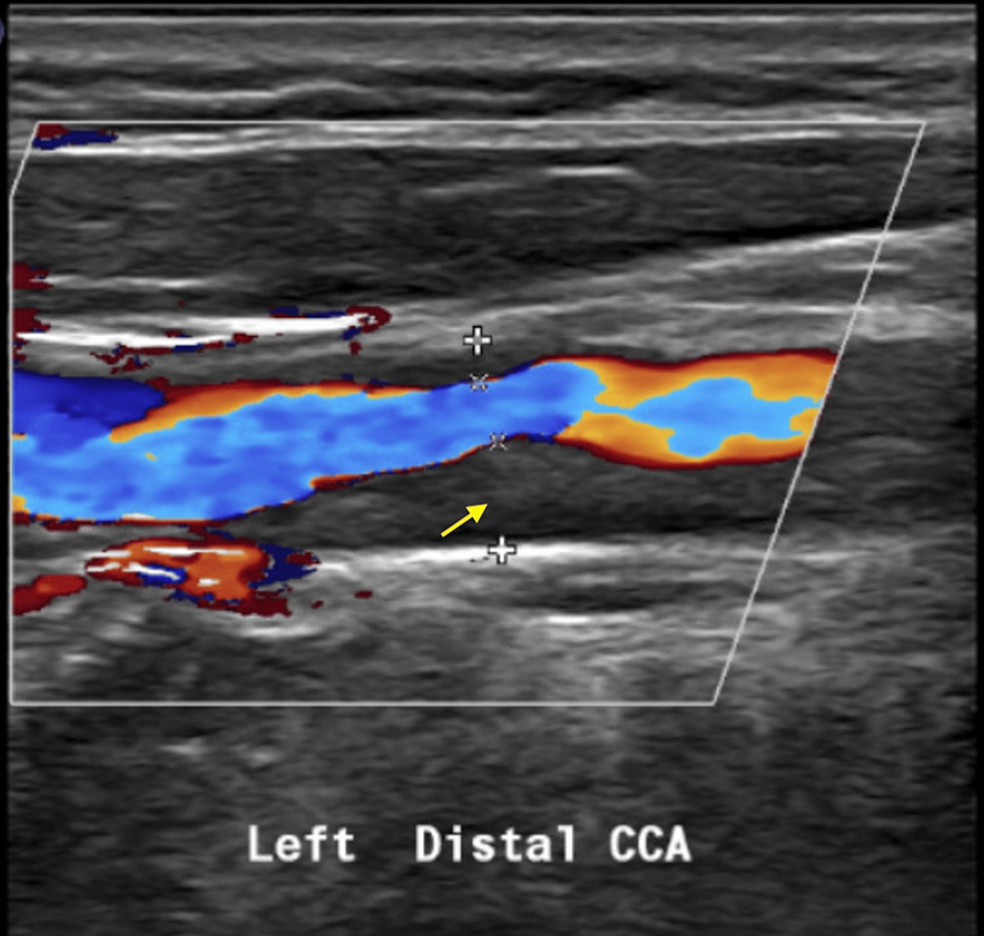
Duplex showing left distal CCA stenosis (yellow arrow) before CEA. CCA: Common carotid artery; CEA: carotid endarterectomy.

**Figure 2:**
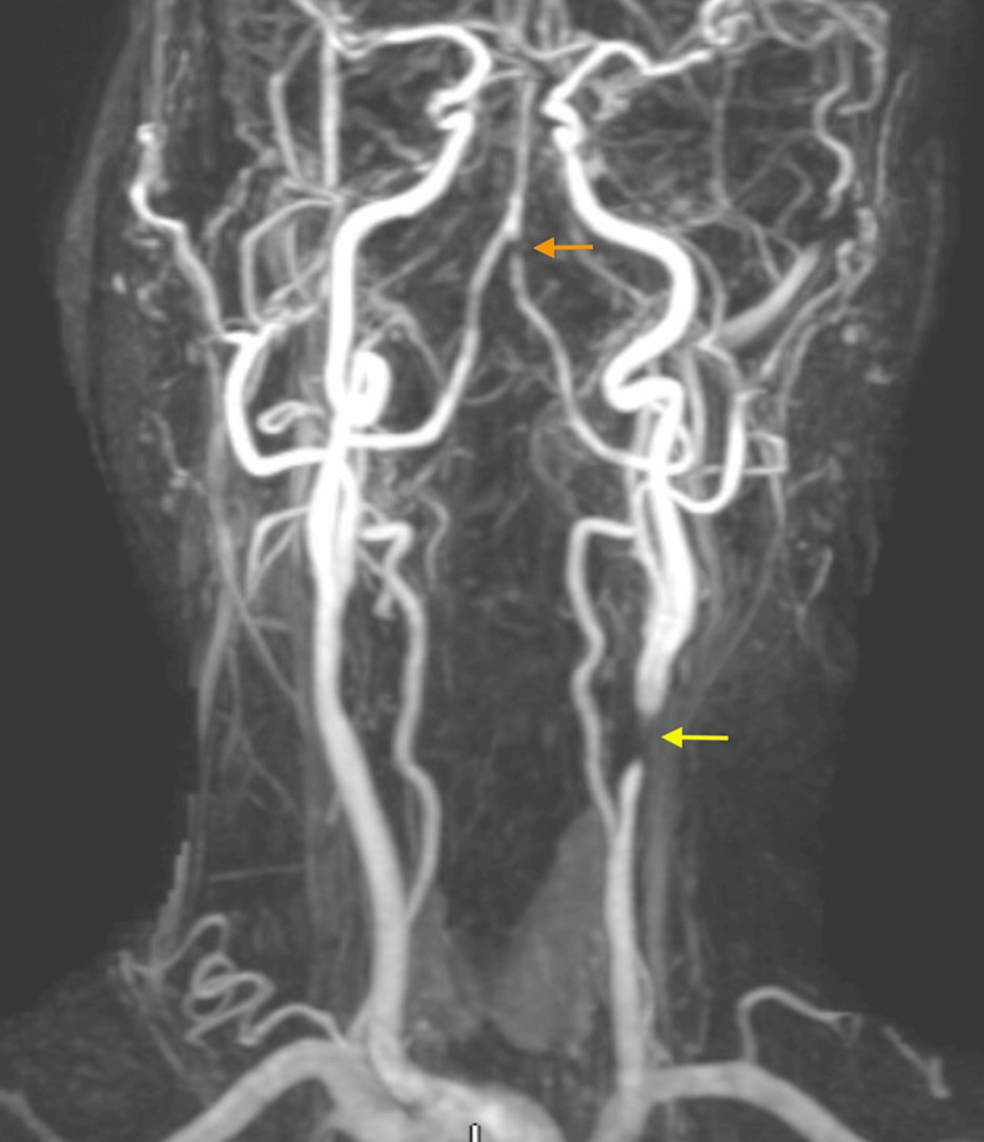
MRA showing left distal CCA stenosis (yellow arrow) and V4 stenosis (orange arrow) before CEA. MRA: magnetic resonance angiography, CCA: common carotid artery, V4: fourth portion of vertebral artery, CEA: carotid endarterectomy.

The patient is a 60-year-old female with a past medical history of type 2 diabetes mellitus (T2DM), hypertension, hyperlipidemia, body mass index (BMI) of 28, and a lifelong non-smoker who presented to the emergency department with acute-onset right-sided weakness and hemiparesis, along with dysarthria. The initial CT scan showed a small area of possible left frontal lobe ischemia with resolution on a short-interval follow-up CT. An MRI subsequently showed a left pontine infarct, and the patient was admitted for management of a cerebrovascular accident (CVA) with primarily dual antiplatelet therapy (DAPT). A carotid artery duplex was performed (Figure 1) and noted distal left CCA peak systolic velocity (PSV) of 426 cm/s, pre-stenotic PSV of 58 cm/s, and end-diastolic velocity (EDV) of 183 cm/s at maximal stenosis, indicative of >75% stenosis. The left ICA had no plaque, PSV of 36 cm/s and a monophasic waveform with a prolonged upstroke, consistent with flow-reducing stenosis of the distal left CCA. A magnetic resonance angiogram (MRA) was also performed during her stroke evaluation and noted similar findings of severe stenosis of the left CCA approximately 1.2 cm from the bifurcation. The ICA and bifurcation were noted to be patent, and a short segment stenosis of the distal left vertebral artery (V4) was also seen (Figure 2).

**Figure 3:**
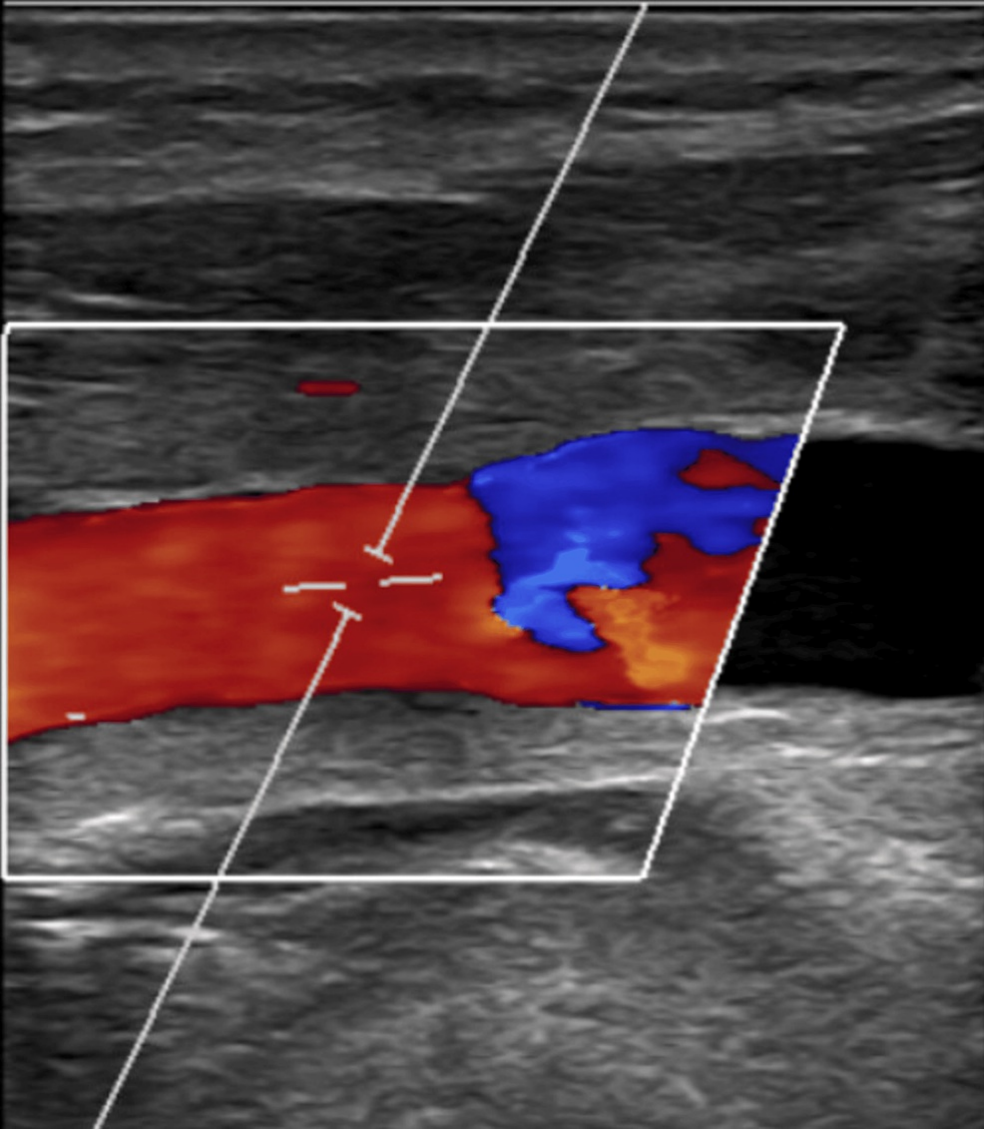
Duplex of left distal CCA after CEA. CCA: common carotid artery, CEA: carotid endarterectomy.

**Figure 4:**
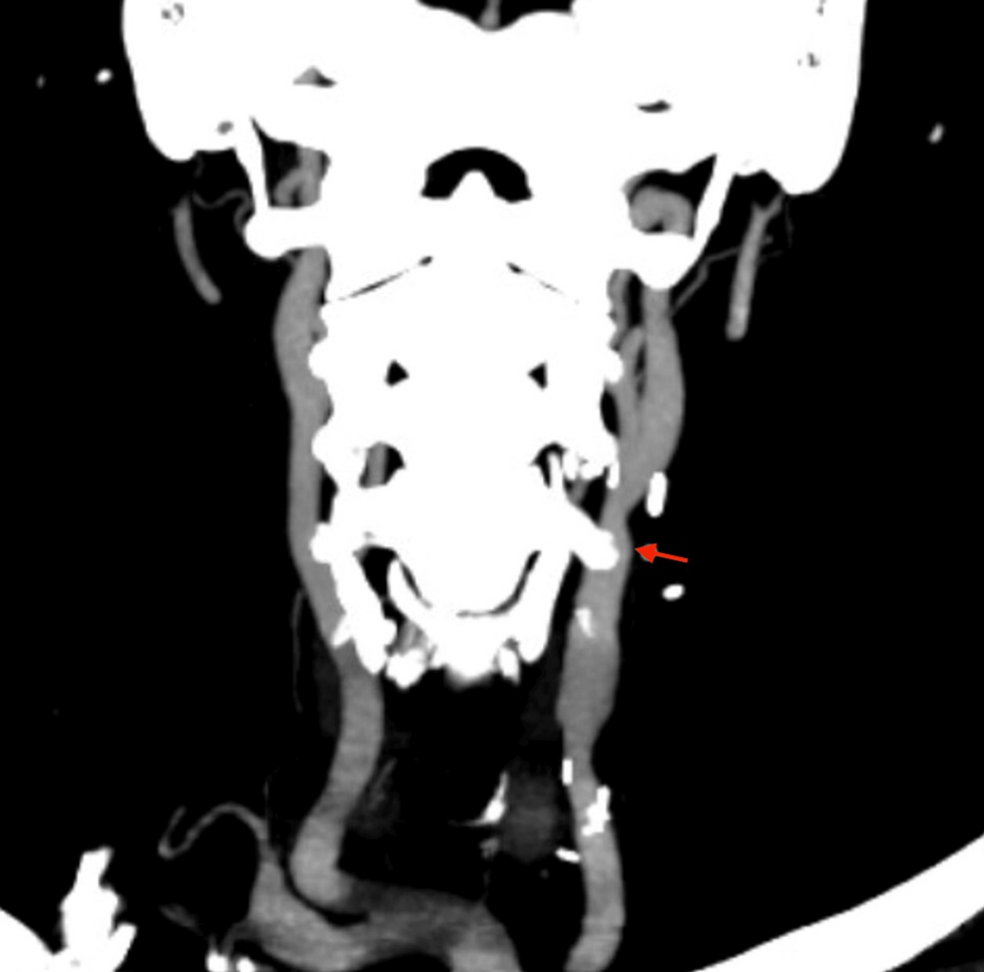
CTA showing patent left distal CCA (red arrow) after CEA. CTA: computed tomography angiography, CCA: common carotid artery, CEA: carotid endarterectomy.

An embolic workup, including an electrocardiogram (ECG), transthoracic echocardiogram (TTE), and computed tomography angiogram (CTA) of the chest, was performed and found to be negative with no evidence of arrhythmia, cardiac abnormalities, or other pathology identified. After stabilization of her condition following concomitant anterior and posterior ischemic events with the return to baseline function, we opted to perform an elective left CCA endarterectomy to address the affected anterior circulation. Of note, the patient had no history of radiation, trauma, or prior surgery to the neck. Approximately three weeks after symptom onset, the CEA was performed in standard fashion as follows. A 10 cm incision along the anterior aspect of the sternocleidomastoid muscle was made and dissection carried down to the carotid sheath, which was opened and carotid bifurcation exposed. We obtained control of the ICA, CCA, and external carotid artery (ECA), which were clamped in sequence before making a 3 cm arteriotomy over the CCA lesion. A moderate plaque was excised, and a shunt was used during the clamping of the ICA due to the distal location of the CCA lesion, for a duration of approximately 38 minutes. Closure of the arteriotomy was performed using a bovine pericardial patch, and clamps were released in retrograde sequence with the removal of the shunt prior to patch completion. The patient was discharged appropriately after the procedure with no complications. There was significant symptom resolution noted on postoperative visits, with no evidence of neurologic deficits detected at one-, three-, or six-month follow-up with no significant changes in the physical exam, and the National Institutes of Health Stroke Scale/Score (NIHSS) consistently remaining at '0', indicating optimal results. A carotid duplex performed at a one-month follow-up (Figure 3) demonstrated a widely patent CCA, ICA and bifurcation, with an ICA/CCA PSV ratio of 0.74. A CTA later obtained at a six-month follow-up (Figure 4) demonstrated similar findings of a widely patent CCA, ICA and bifurcation. The patient has agreed to have his case published and written consent obtained.

## Discussion

The authors present a case of isolated CCA stenosis presenting with neurologic symptoms atypical of lesions at this location, successfully managed with a CEA with reassuring postoperative outcomes.

Atherosclerosis preferentially develops at the bifurcation of the CCA into the ICA, with proximal CCA disease infrequently described in the literature [6]. Symptomatic presentation of CCA occlusion (CCAO) or clinically significant stenosis is uncommon, as the external carotid artery (ECA) may be perfused in a retrograde manner and thus maintain anterograde flow within the ICA [7,8]. Due to its rarity, scant recommendations exist for the treatment of symptomatic CCA stenosis with no consensus statements in the clinical practice guidelines from either the Society for Vascular Surgery (2022) [2] or the American Heart Association (2011) [9]. The closest approximation to the recommendation exists within the new clinical practice guidelines from the European Society for Vascular Surgery (ESVS) (2023) [10], which “cautiously support” endovascular first strategies for isolated symptomatic CCA stenosis; however, based on severely limited research. Although not a consensus statement, an executive summary from several cerebrovascular societies and experts in the field suggests that percutaneous angioplasty and carotid artery stenting (CAS), direct arterial reconstruction, or extra-anatomic bypass may be reasonable interventions to treat symptomatic disease caused by CCA lesions [9]. In clinical practice, without formal standards of care for this disease entity, treatment is often individualized in accordance with the location of the lesion, the feasibility of the proposed intervention, or the surgeon's preference.

Retrograde CCA stenting (RCCAS) is a very well-described procedure that is often performed for stenosis or occlusion of the innominate artery or the CCA [11]. This may be accomplished as an isolated endovascular procedure or in conjunction with CEA to achieve the patency of the CCA [12,13]. This technique may also satisfy the recommendations provided by the ESVS advocating for an endovascular approach as a first option. In our presented case, however, this technique is not feasible due to the distal CCA location of this stenosis (1.2 cm from bifurcation). This procedure would require cannulation of the CCA distal to the lesion, but with such a short segment between the lesion and the bifurcation, the only access could be obtained with cannulation of the ICA, which would significantly increase stroke risk. Vertes et al. (2020) [14] acknowledge this shortcoming and advocate for a preferential antegrade approach. Aside from these factors, due to the desirable health of this patient and no preclusions to open surgery, we elected to proceed with a CEA.

Within the literature, there are several (albeit few) alternative treatment options described that may be employed for patients with symptomatic CCA stenosis. These are as follows: Saito et al. (2019) [6] reported a case of proximal CCA stenosis caused by repetitive extrinsic compression. This patient was initially treated with medical management, utilizing DAPT. This proved unsuccessful, as four months later, the patient presented with recurrent symptoms and worsening stenosis, and subsequently underwent successful CAS of the CCA with an unremarkable ensuing postoperative course. In 2018, Wang et al. [15] performed a retrospective review of 39 patients with long-segment, symptomatic, chronic CCA occlusive disease who underwent in-situ ring-stripper-mediated retrograde endarterectomy with a 100% technical success rate, with symptomatic improvement in all patients with no postoperative complications over 2.5 years. Other alternative treatments proposed for poor candidates for CAS include subclavian artery-CCA or ICA bypass, as demonstrated in a case series by Yamaguchi et al. (2023) [16], with no symptom recurrence and good postoperative outcomes during a two-year follow-up. This option is supported by the largest literature review performed to date of CCAO management [4], which identifies 21 articles encompassing 146 patients with symptomatic disease. In patient undergoing anatomic bypass, these review articles demonstrate desirable endpoints of procedural safety with low perioperative morbidity and optimal postoperative outcomes. 

## Conclusions

CCAO and stenosis, while less common than disease at the bifurcation and ICA, remain clinically relevant pathologies, and current societal recommendations for ICA disease may not reflect optimum treatment for this specific location. Although uncommon relative to the typical presentation of carotid disease, a literature review notes increasing encounters with symptomatic CCA lesions. Providers continue to document their management strategies, and there appears to be increased attention drawn to the need for specific guidelines for CCAO, as evidenced by recent ESVS consensus statements. Continued research and larger studies on CCA disease are essential to address this gap in the literature, and to accommodate specific management recommendations in future societal guidelines.
